# Identification and Characterization of Cannabimovone, a Cannabinoid from *Cannabis sativa*, as a Novel PPARγ Agonist via a Combined Computational and Functional Study

**DOI:** 10.3390/molecules25051119

**Published:** 2020-03-03

**Authors:** Fabio Arturo Iannotti, Fabrizia De Maio, Elisabetta Panza, Giovanni Appendino, Orazio Taglialatela-Scafati, Luciano De Petrocellis, Pietro Amodeo, Rosa Maria Vitale

**Affiliations:** 1 Institute of Biomolecular Chemistry (ICB), National Research Council (CNR), 80078 Pozzuoli (NA), Italy; 2 Endocannabinoid Research Group (ERG), Institute of Biomolecular Chemistry, National Research Council (ICB-CNR), Via Campi Flegrei 34, 80078 Pozzuoli (NA), Italy; 3 Department of Pharmacy, University of Naples “Federico II”, Via D. Montesano 49, I-80131 Napoli, Italy; 4 Dipartimento di Scienze del Farmaco, Università del Piemonte Orientale, Largo Donegani 2, 28100 Novara, Italy

**Keywords:** phytocannabinoids, cannabimovone (CBM), peroxisome proliferator-activated receptor gamma (PPARγ), molecular docking, molecular dynamics, insulin resistance

## Abstract

Phytocannabinoids (pCBs) are a large family of meroterpenoids isolated from the plant *Cannabis sativa*. Δ^9^-Tetrahydrocannabinol (THC) and cannabidiol (CBD) are the best investigated phytocannabinoids due to their relative abundance and interesting bioactivity profiles. In addition to various targets, THC and CBD are also well-known agonists of peroxisome proliferator-activated receptor gamma (PPARγ), a nuclear receptor involved in energy homeostasis and lipid metabolism. In the search of new pCBs potentially acting as PPARγ agonists, we identified cannabimovone (CBM), a structurally unique *abeo*-menthane pCB, as a novel PPARγ modulator via a combined computational and experimental approach. The ability of CBM to act as dual PPARγ/α agonist was also evaluated. Computational studies suggested a different binding mode toward the two isoforms, with the compound able to recapitulate the pattern of H-bonds of a canonical agonist only in the case of PPARγ. Luciferase assays confirmed the computational results, showing a selective activation of PPARγ by CBM in the low micromolar range. CBM promoted the expression of PPARγ target genes regulating the adipocyte differentiation and prevented palmitate-induced insulin signaling impairment. Altogether, these results candidate CBM as a novel bioactive compound potentially useful for the treatment of insulin resistance-related disorders.

## 1. Introduction

Peroxisome proliferation-activated receptors (PPARs) are ligand-activated transcription factors involved in the regulation of energy homeostasis and metabolism [1]. The binding of agonists to the PPAR ligand-binding domain (LBD) induces conformational changes, leading to the release of a corepressor and the recruitment of coactivator peptides. The LBD is a 12-helix bundle domain with a central core forming the ligand binding pocket. The inactive conformation is characterized by high flexibility of the short helix H12, part of the coregulator interaction surface (AF-2), along with helices H3 and H11, and the H3–H4 loop [2]. Ligand binding stabilizes H12, triggering the switch toward the PPAR’s active conformation. After binding to the agonist, PPARs heterodimerize with another nuclear receptor, the retinoid X receptor (RXR), to modulate the transcription of its target genes [3]. Three different PPAR isoforms, namely α, γ, and β/δ, were characterized so far [4], with PPARγ playing a crucial role in regulating adipocyte differentiation, lipid metabolism, glucose homeostasis, and insulin resistance [5]. For these reasons, it represents a pharmacological target for the treatment of insulin resistance and dyslipidemia [6,7]. However, the therapeutic use of potent PPARγ agonists such as thiazolidinediones is restricted due to their severe side effects including weight gain, fluid retention, edema, and myocardial infarction [8,9]. Thus, a promising strategy consists of the development of less potent PPARγ modulators endowed with an acceptable safety profile. In this view, natural products represent an invaluable source of bioactive molecules due to their high chemical diversity. Among them, phytocannabinoids (pCBs) deserve special interest, since their major compounds, Δ^9^-tetrahydrocannabinol (THC) and cannabidiol (CBD), show a rich polypharmacology [10,11,12,13] including PPARγ agonism [14,15]. However, when considering the diversity of the chemical space of phytocannabinoids [16], their pharmacological potential as PPARγ is still substantially untapped. In this context, we focused our attention on cannabimovone (CBM), a pCB from fiber hemp (*Cannabis sativa* variety “Carmagnola”) with a unique *abeo*-menthane terpenoid skeleton substantially different from that of THC and CBD [17], scouting its potency to act as a PPARγ agonist via molecular docking and molecular dynamics simulations, followed by validating the results with cellular assays. The PPARα isoform was also evaluated to identify a possible dual profile of PPARα/γ modulation.

## 2. Results

### 2.1. Molecular Docking and Molecular Dynamics

**Figure 1:**
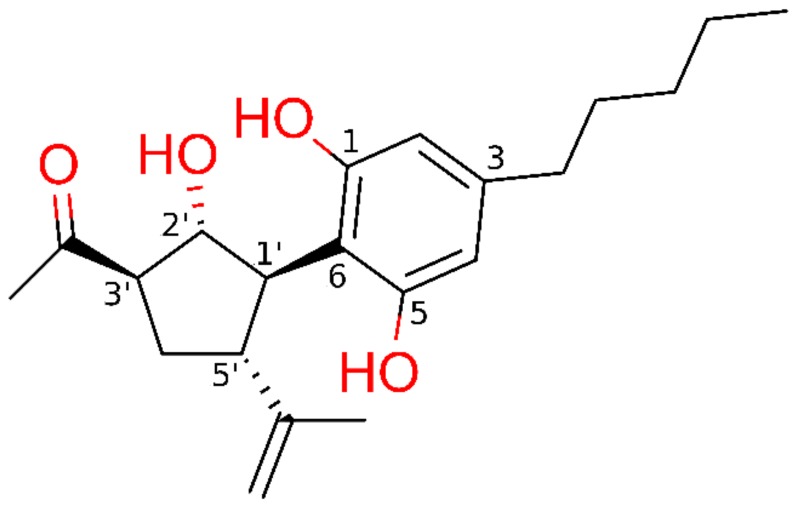
Chemical structure of cannabimovone (CBM) with functional groups colored in red.

The potential ability of cannabimovone (CBM, Figure 1) to bind and activate PPARγ and/or PPARα was investigated via a computational approach based on a combination of molecular docking and molecular dynamics. For each receptor, the docking poses representative of the most populated clusters endowed with the most favorable binding energy were selected to undergo 50-ns molecular dynamics (MD) simulations to assess their stability, performing the MD analysis on the last 45 ns. Unless differently stated, only the H-bonds with an occurrence >10% during MD are reported.

#### 2.1.1. Theoretical Complexes of PPARγ with Cannabimovone (CBM)

**Figure 2:**
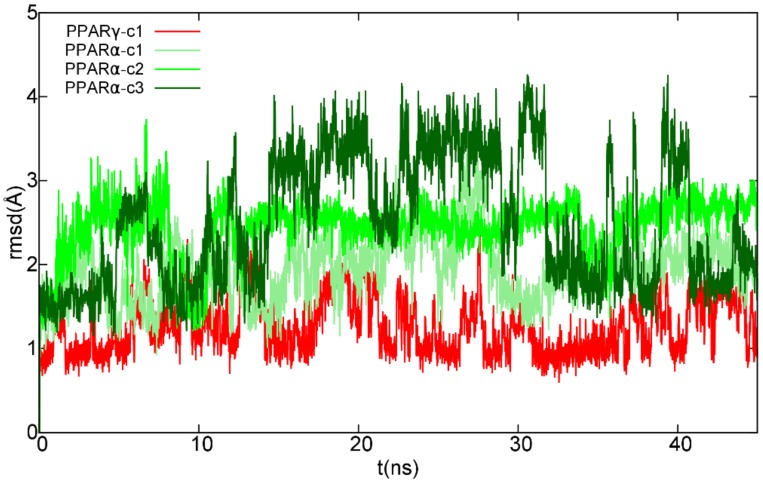
Root-mean-square deviation (RMSD) plot of CBM in complex with peroxisome proliferation-activated receptor (PPAR) γ/α over the last 45 ns of molecular dynamics (MD) trajectory after best fitting of protein backbones. The ligand RMSD plot was smoothed with a five-point window running average. Red color is used for CBM in complex with PPARγ, while different shades of green are used for the ligand in the three simulated PPARα complexes.

**Figure 3:**
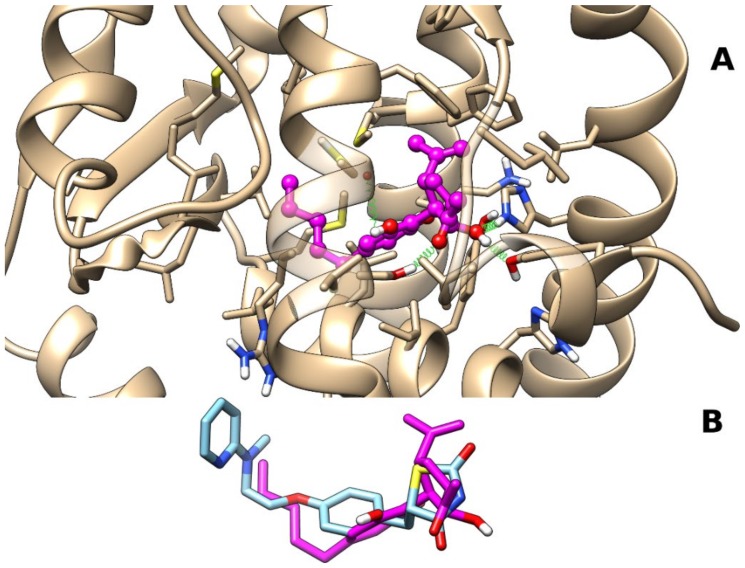
Representative frame from MD of PPARγ–CBM complex. Panel (**A**) A ball-and-stick representation is used for heavy atoms of the ligand, and a stick representation is used for protein sidechains within 5 Å of the ligand. Protein carbon atoms are colored in tan according to the ribbon for the protein and in magenta for CBM. Hydrogen, nitrogen, oxygen, and sulfur atoms are painted white, blue, red, and yellow, respectively. Half-transparency is employed for the ribbon representation of protein regions overlying the ligand in the selected view. A “green spring” representation is adopted for H-bonds involving ligand atoms. Panel (**B**) Stick representation of CBM and rosiglitazone (Protein Data Bank (PDB) identifier (ID): 5ycp) after best fit of protein backbone. Carbon atoms of rosiglitazone are colored in light blue, while heteroatoms are colored according to panel (**A**).

The analysis of docking results showed the occurrence of a single most populated cluster characterized by the best binding energy. The representative pose from the cluster was chosen to undergo the subsequent molecular dynamics (MD) simulation. After a slight initial rearrangement of the ligand alkyl chain, the complex remained stable through the last 45 ns (see Figure 2). The representative frame from MD, selected as described in Section 4, is reported in Figure 3. The polar groups of CBM engage stable H-bonds with Tyr473 (H12), a residue critical for PPARγ activation, along with His449 (H10–11) and Ser289 (H3). In detail, the CBM carbonyl group engages an H-bond with the hydroxyl group of Ser289 (H3) (occurrence of 66%), the CBM hydroxyl group in 2′ forms an H-bond network with both Tyr473 (H12) (occurrence of 75%) and His449 (H10–11) (occurrence of 52%) sidechains, and the CBM hydroxyl group in 1 forms H-bonds alternatively with the backbone carbonyl of Cys285 (H3) (occurrence of 49%) or Ser289 (H3) sidechain (occurrence of 17%). The isopropenyl group forms van der Waals interactions with both Phe282 (H3) and Phe363 (H7), while the pentyl chain interacts with Val339 in the β-sheet.

#### 2.1.2. Theoretical Complexes of PPARα with Cannabimovone (CBM)

**Figure 4:**
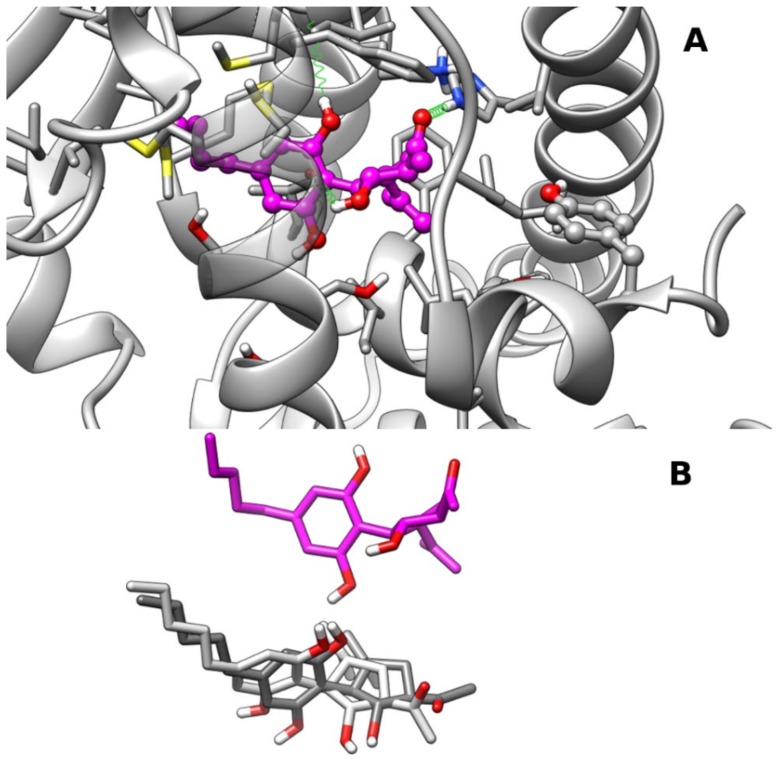
Representative frames from MD of PPARα–CBM complexes: Panel (**A**) A ball-and-stick representation is used for the heavy atoms of the ligand, and a stick representation is used for the protein sidechain within 5 Å of the ligand. Protein carbon atoms are colored in dark gray according to ribbon for protein and in magenta for CBM. Hydrogen, nitrogen, oxygen, and sulfur atom are painted white, blue, red, and yellow, respectively. Half-transparency is employed for the ribbon representation of protein regions overlying ligands in the selected view. A “green spring” representation is adopted for H-bonds involving ligand atoms. Panel (**B**) Stick representation of CBM poses in clust2 (light gray), clust3 (dark gray), and clust1 (magenta) after best fitting of protein backbone. CBM pose in clust1 was translated vertically for clarity.

The cluster analysis of docking results showed more dispersed binding poses in comparison with PPARγ, with the three most populated clusters within 0.4 kcal/mol of binding energy. The representative frames from MD of each cluster complex are shown in Figure 3 and are hereinafter referred to as clust1, clust2, and clust3. Clust2 and clust3 show a very similar overall arrangement of the ligand in the LBD (see Figure 4B), but differ from each other in the orientation of the carbonyl group, whereas clust1 is shifted more toward helices H7/H10–11 (see Figure 4A,B). In all poses, the five-membered ring is tilted compared to the pose adopted in PPARγ, possibly due to the difference in steric hindrance of Ile354 (H7) in place of PPARγ Phe363 and Tyr314(H5) in place of PPARγ His323 (H5), respectively. In both clust1 and clust2, the CBM carbonyl oxygen engages an H-bond with His440 (H10/11) with an occurrence of 71% and 48%, respectively, but not with Tyr464 (H12) as in the case of the hydroxyl group in 2′ in PPARγ. In clust3, CBM adopts a similar arrangement to clust2 but it is shifted more toward the β-sheet, and the carbonyl group no longer forms an H-bond with His440 (H10/11), but instead forms one with the sidechain of Ser280 (H3) (occurrence of 25%). In clust1, the CBM hydroxyl group in 1 forms H-bonds alternatively with the backbone carbonyl of Cys276 (H3) (occurrence of 48%) and Ser280 (H3) sidechain (occurrence of 21%), while the hydroxyl group in 2 engages an H-bond with the backbone carbonyl of Ile354 (H7) (occurrence of 35%). Clust2 and clust3 share with clust1 the formation of an H-bond between the hydroxyl group in 1 and the backbone carbonyl of Cys276 (H3) with an occurrence of 53% and 33%, respectively.

### 2.2. Luciferase Assays

**Figure 5:**
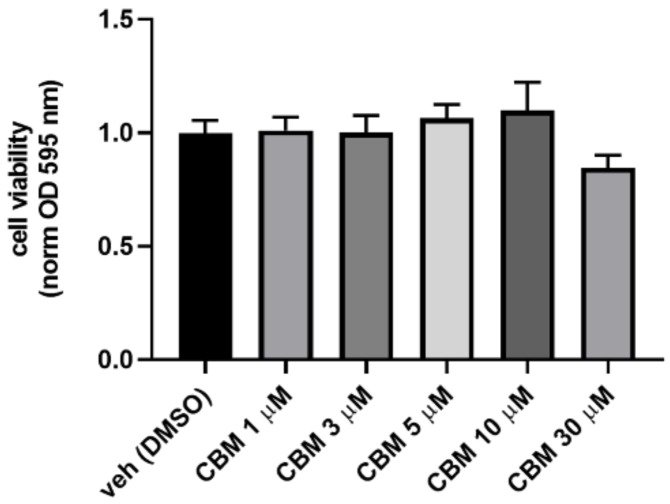
Cell viability measured in HEK293 cells treated for 24 h with crescent concentration of CBM. Bar graph shows cell viability measured using the 3-(4,5-dimethylthiazol-2-yl)-2,5-diphenyltetrazolium bromide (MTT) assay. Data are expressed as optical density (OD) at 595 nm, normalized to control (vehicle). Each point is the mean ± standard error of the mean (SEM) of four separate determinations performed in duplicate. Statistically significant differences were accepted when the *p*-value was at least ≤0.05.

**Figure 6:**
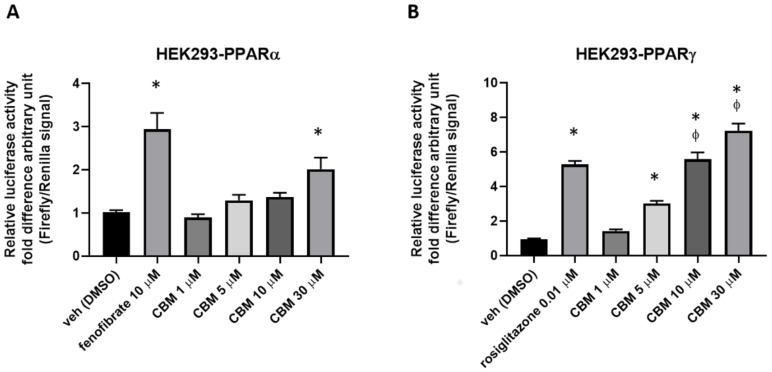
Luciferase assays performed in PPARα- (A) or PPARγ-transfected (B) HEK293 cells. Bar graphs showing the ratio between firefly and *Renilla* luciferase in response to crescent concentrations of cannabimovone (CBM; up to 30 μM). Fenofibrate and rosiglitazone were used as positive controls for PPARα and PPARγ, respectively. The vehicle group was set to 1; thus, the relative luciferase activities obtained for each tested compound and concentration are presented as a fold induction with respect to the vehicle control. Each point is the mean ± SEM of four separate determinations performed in duplicate. Statistically significant differences were accepted when the *p*-value was at least ≤0.05. The asterisk (*) denotes a *p*-value ≤ 0.05 vs. vehicle group; the symbol ϕ denotes *p* ≤ 0.05 vs. vehicle group vs. CBM 5 µM.

To evaluate the potential cytotoxicity of CBM at the concentrations used for our functional assays, preliminary experiments of cell viability using the 3-(4,5-dimethylthiazol-2-yl)-2,5-diphenyltetrazolium bromide (MTT) assay were carried out on human embryonic kidney 293 (HEK293) cells. Therefore, HEK293 cells were exposed to increasing concentrations of CBM (1, 5, 10, and 30 µM), and the results shown in Figure 5 demonstrate that CBM does not cause significant cytotoxic effects at any tested concentration. Subsequently, HEK293 cells were transiently transfected with the chimeric human PPARα-LBD-Gal4 or PPARγ-LBD-Gal4 constructs concomitantly to a UAS enhancer (MH100). The day after transfection, cells were treated with CBM at 1, 5, 10, and 30 µM. Fenofibrate (10 µM) and rosiglitazione (0.01 µM) were used as positive controls for PPARα and PPARγ, respectively. As shown in Figure 6A,B, CBM promoted transcriptional activity in PPARα-transfected cells only at the highest assayed concentration (30 µM). In contrast, the effect of CBM on PPARγ activity was much more prominent and concentration-dependent.

### 2.3. CBM Upregulates Genes Involved in Adipocyte Differentiation and Energy Metabolism

**Figure 7:**
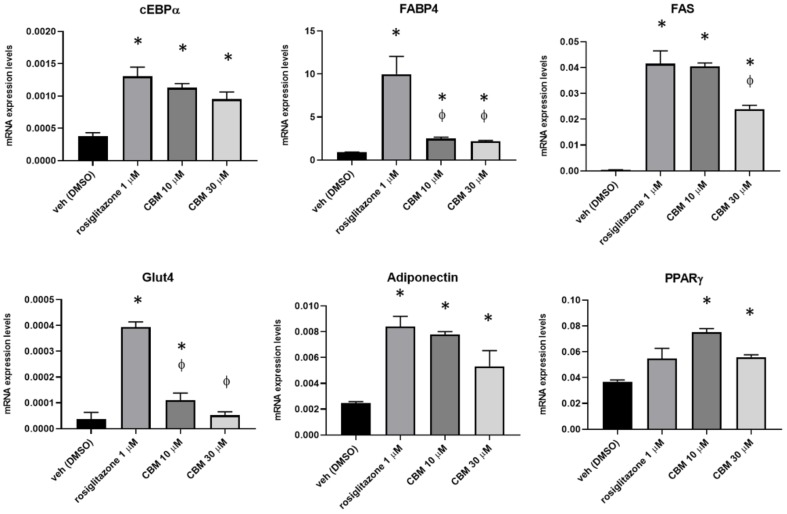
Effect of rosiglitazone and CBM in differentiating 3T3-L1 cells. Transcript levels of CCAAT/enhancer binding protein alpha (C/EBPα), fatty acid- binding protein 4 (FABP4), glucose transporter 4 (GLUT4), fatty acid synthase (FAS), adiponectin, and PPARγ in murine 3T3-L1 cells exposed to differentiation media (DM) in the presence of vehicle (dimethyl sulfoxide (DMSO)), rosiglitazone 1 µM, or CBM 10 and 30 µM. The quantification of transcripts was performed by qPCR. Data represent the mean ± SEM of four independent determinations. Data are expressed as 2^−^Δct^ relative to S16, as described in Section 4. Datasets were compared with one-way ANOVA followed by Tukey’s test. Differences were considered statistically significant when *p* ≤ 0.05. The asterisk * denotes a *p*-value ≤ 0.05 vs. vehicle group; the symbol ϕ denotes *p* ≤ 0.05 vs. vehicle group vs. CBM 5 µM.

It is well known that, once activated by a ligand, PPARγ receptors bind to specific PPAR response elements (PPRE) to control the transcriptional activity of an array of genes, orchestrating a plethora of metabolic responses that impact the regulation of insulin sensitivity, as well as lipid and cholesterol metabolism. Furthermore, PPARγ activity is also important to trigger the differentiation and function of pre-adipocytes [18,19]. Therefore, to further explore the pharmacological activity of CBM on PPARγ, we induced 3T3-L1 pre-adipocytes to differentiate for 10 days in the presence of rosiglitazone at 1 µM, and CBM at both 10 and 30 µM, to further explore the pharmacological activity of CBM on PPARγ. After this time, the total RNA was isolated from each experimental condition and the expression of transcripts encoding for key PPARγ-regulated gene markers of adipogenesis, including CCAAT/enhancer binding protein alpha (C/EBPα), fatty acid-binding protein 4 (FABP4), glucose transporter 4 (GLUT4), fatty acid synthase (FAS), and adiponectin [20,21], was measured by quantitative PCR (qPCR). The data revealed that, in 3T3-L1 cells induced to differentiate in the presence of rosiglitazone, as expected, the expression of all the mature adipocytes markers was robustly increased compared to the control group (Figure 7). In CBM-treated cells we observed that the increase in the expression of cEBPα, adiponectin, and FAS (only at 10 µM) was comparable to that observed with rosiglitazione (Figure 7). Less robust but still evident when compared to the control group was the expression of FABP4 and GLUT4. It is noteworthy that CBM, but not rosiglitazone, significantly increased the expression of PPARγ, revealing that, most likely, this phytocannabinoid not only activates PPARγ, but it is also able to trigger a positive feedback loop promoting gene expression.

### 2.4. CBM Improves Insulin Sensitivity in Differentiating 3T3-L1 cells

**Figure 8:**
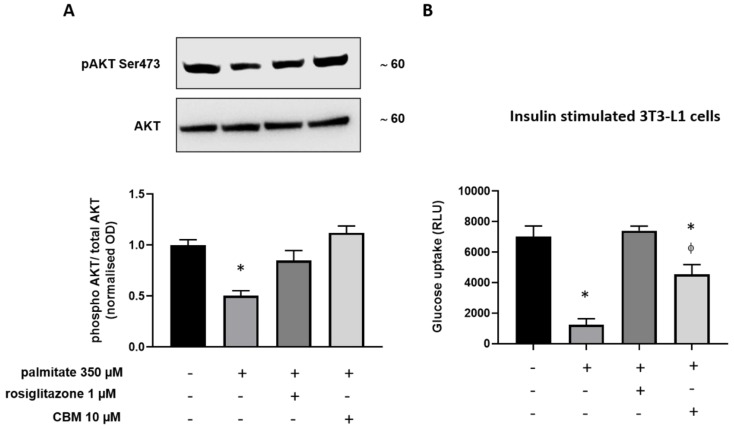
Measurement of protein kinase B (Akt) phosphorylation in differentiated 3T3-L1 cells exposed to sodium palmitate (NaP) in the presence or absence of rosiglitazone and CBM. (**A**) Upper representative blot showing the band intensity of phospho-(Ser473) and total Akt in differentiated 3T3-L1 cells treated with sodium palmitate 350 µM for 18 h. Lower bar graph showing the quantification of phospho-Akt levels normalized to Akt (total). (**B**) Levels of glucose uptake expressed as relative luminescence units (RLU) in insulin-stimulated 3T3-L1 cells. Data represent the mean ± SEM of three separate determinations. Datasets were compared by one-way ANOVA followed by Tukey’s test. Differences were considered statistically significant when *p* ≤ 0.05. The asterisk (*) denotes a *p*-value ≤ 0.05 vs. vehicle control group (left) or stimulated 3T3-L1 cells with insulin 100 nM (right); the symbol ϕ denotes a *p*-value ≤ 0.05 vs. rosiglitazone.

Finally, we explored whether, in differentiated 3T3-L1 cells, the insulin signaling impairment induced by palmitate could be prevented by CBM via PPARγ activation. In this view, 3T3-L1 cells were firstly induced to differentiate for 10 days. Subsequently, the cells were incubated in fresh differentiation media in the presence of rosiglitazone 0.01 µM or CBM 10 µM. After 2 h, sodium palmitate (NaP 350 µM) was added to each cell plate and incubated for a further 18 h [22]. As shown in Figure 8A, we found that, in differentiated 3T3-L1 cells, palmitate significantly reduced the phosphorylation of protein kinase B (Akt), physiologically required for insulin-mediated glucose uptake [23]. However, in the presence of rosiglitazone and CBM, the phosphorylation of Akt was fully recovered. Along with this, we measured the glucose uptake under the same experimental conditions. As shown in Figure 8B, palmitate significantly inhibited (100 nM) insulin-evoked uptake of glucose. This latter effect was fully prevented by rosiglitazone and also significantly prevented by CBM 10 µM. CBM 30 µM produced no further effects (data not shown).

## 3. Discussion

Phytocannabinoids (pCBs) are a class of secondary metabolites isolated from *C. sativa*, counting more than 100 compounds with a still largely unexplored pharmacological potential [16]. THC and CBD, the most representative pCBs, have interesting therapeutic applications due to their complex pharmacological profile, and they are active on the PPARγ receptor, while a few other neutral and acidic pCBs were characterized as dual PPARα/γ agonists [15]. Due to its role in adipocyte differentiation, lipid metabolism, glucose homeostasis, insulin sensitivity, and, more recently, in inflammatory and immune responses, PPARγ represents an attractive pharmacological target to address metabolic disorders. As part of our discovery program on nuclear receptor ligands from marine [24,25] and terrestrial sources [15], we focused on cannabimovone (CBM), a polar pCB characterized by a unique *abeo*-menthane terpenyl moiety isolated in 2010 from a non-psychotropic variety of *C. sativa* (Italian cultivar Carmagnola) [17]. The chemical structure of cannabimovone, including stereochemical details, was recently confirmed by total synthesis, which can also be considered as an alternative to the exploitation of the natural source [26]. The scarcity of pharmacological data available on CBM prompted us to investigate in silico the suitability of this scaffold to act as a potential selective or dual PPARα/γ agonist. From docking and molecular dynamics studies, a difference in the binding modes between the two isoforms emerged, suggesting a stronger activity toward PPARγ in comparison to PPARα. In fact, while, in the PPARγ complex, CBM is able to directly interact through H-bond with Tyr473 (H12), the key residue located on the AF-2 domain and responsible for the activation of the receptor by full agonists, in the PPARα complex, a different arrangement of the ligand was observed, with the lack of the aforementioned interaction. Moreover, in all the simulated PPARα complexes, CBM is characterized by a higher mobility than in the PPARγ complex, as shown in the root-mean-square deviation (RMSD) plot (Figure 2). To validate the computational results, luciferase assays on both PPARγ and PPARα were carried out, confirming the higher potency of CBM toward PPARγ in comparison to PPARα (Figure 6) with a dose-dependent activation of Gal4-PPARγ in the former case. However, when compared to rosiglitazone, CBM is a weaker PPARγ agonist, and this can be mainly ascribed to its reduced size, which prevents the full occupancy of the active site. In particular, the alkyl chain has only a limited interaction with the β-sheet region of the LDB (see Figure 3), usually deeply involved in hydrophobic interactions with potent PPARγ agonists. However, in principle, a lower potency should be associated with a better safety profile. In this view, the cell viability assay (MTT) assay showed no-toxicity of CBM up to 30 μM, while, for other phytocannabinoids, we found a toxic effect even at 10 μM [15]. Then, we further evaluated the effect of CBM on PPARγ downstream genes involved in adipocyte differentiation, such as PPARγ, C/EBPα, and FAPB4. Our results indicate that CBM promotes adipocyte differentiation in 3T3-L1 by increasing PPARγ transcriptional activity at the aforementioned genes. Adipose tissue insulin resistance is characterized by a deficiency in GLUT4 [27], and rosiglitazone, a well-known insulin-sensitizer, acts at least in part by enhancing GLUT4 expression [28]. In the present study, we found that also CBM upregulates GLUT4 and adiponectin expression, thus promoting insulin-stimulated glucose transport in 3T3-L1 adipocytes. Moreover, since numerous studies suggest that the phosphorylation-mediated activation of Akt, which in turn positively regulates downstream targets including Glut4, glycogen synthase kinase 3 (GSK3), forkhead box O1 (Foxo1), hormone sensitive lipase (HSL), and mammalian target of rapamycin (mTOR), is one of the most important intracellular pathways promoting insulin sensitivity [29,30,31], we evaluated the effect of CBM on the insulin signaling impairment mediated by palmitate. In our study, CBM fully restored the reduced levels of Akt phosphorylation induced by palmitate, as well as increased the level of insulin-mediated glucose uptake (Figure 8A,B). In conclusion, our combined computational and experimental approach led to the identification and a structural and functional characterization of a novel phytocannabinoid as a PPARγ agonist able to in vitro stimulate insulin signaling, paving the way for further in vivo studies to assess the suitability of CBM as anti-diabetic and insulin-sensitizing drug, thus opening new therapeutic alternatives for those patients still not receiving an effective and safe long-term treatment.

## 4. Methods

### 4.1. Purification of Cannabimovone

CBM used in tests was obtained from the polar fraction of the acetone extract of a non-psychotropic variety of *C. sativa*. Isolation, purification, and structural elucidation of CBM were described elsewhere [17].

### 4.2. Molecular Docking and Molecular Dynamics

Starting ligand geometry was built with Ghemical 2.99.2 [32] followed by initial energy minimization (EM) at the molecular mechanics level, using Tripos 5.2 force field parametrization [33], and then at the AM1 semi-empirical level. The molecule was then fully optimized using the GAMESS program [34] at the Hartree–Fock level with the STO-3G basis set and subjected to HF/6-31G*/STO-3G single-point calculations to derive the partial atomic charges using the RESP procedure [35]. Docking studies were performed with AutoDock 4.2 [36], by using PPARα and PPARγ crystallographic structures (Protein Data Bank (PDB): 2P54 and 2F4B, respectively). Both proteins and ligands were processed with AutoDock Tools (ADT) package version 1.5.6rc1 [36] to merge non-polar hydrogens and calculate Gasteiger charges. Grids for docking evaluation with a spacing of 0.375 Å and 60 × 60 × 60 points, centered on the ligand binding site, were generated using the program AutoGrid 4.2 included in Autodock 4.2 distribution. The Lamarckian genetic algorithm (LGA) was adopted to perform 100 docking runs with the following parameters: 100 individuals in a population with a maximum of 15 million energy evaluations and a maximum of 37,000 generations, followed by 300 iterations of Solis and Wets local search. The complexes, selected on the basis of binding energy and cluster population, were completed by addition of all hydrogen atoms, and they underwent energy minimization (EM) and then molecular dynamics (MD) simulations with Amber16 pmemd.cuda module [37], using the ff14SB version of the AMBER force field for the protein and gaff parameters [38] for the ligand.

To perform MD simulations in solvent, the complexes were confined in TIP3P water periodic truncated octahedron boxes exhibiting a minimum distance between solute atoms and box surfaces of 10 Å, using the tleap module of the AmberTools16 package. The systems were then neutralized by addition of counterions (Na^+^) and subjected to 1000 steps of EM with solute atoms harmonically restrained to their starting positions (K_r_ = 10 kcal·mol^−1^·Å^−1^). Then, a 500-ps restrained MD simulation (K_r_ = 5 kcal·mol^−1^·Å^−1^) at constant pressure was run on each solvated complex, gradually heating the system to 300 K, followed by a 500-ps restrained MD simulation (K_r_ = 5 kcal·mol^−1^·Å^−1^) at constant temperature (300 K) and pressure (1 atm) to adjust system density. Production MD simulations were carried out at constant temperature (300 K) and pressure (1 atm) for 50 ns, with a time step of 2 fs. Bonds involving hydrogens were constrained using the SHAKE algorithm [39]. The cpptraj module of AmberTools16 and program UCSF Chimera 1.10.1 [40] were used to perform MD analysis and to draw the figures, respectively. Cluster analysis was carried out with the cpptraj module using the dbscan clustering algorithm using the following parameters: minpoints 25, epsilon 0.9, sieve 10. The representative frames were taken from the most populated clusters of each MD simulation.

### 4.3. Cell Culture, Transfection, and Luciferase Assay

Human embryonic kidney 293 cells (HEK293) were propagated in in a growth medium (GM) composed of Dulbecco’s modified Eagle’s medium (DMEM) supplemented with 10% fetal bovine serum and 1% penicillin/streptomycin under standard conditions. After plating, the cells were transfected on the next day with the following plasmids: (a) pM1-hPPARα-Gal4 or pM1-hPPARγ-Gal4, (b) TK-MH100 × 4-Luc containing the UAS enhancer elements, and (c) *Renilla* luciferase (pRL, Promega, Cat. E2231) using lipofectamine 2000 (cat. n. 11668027; Life Technologies; Milan, Italy) following the manufacturer’s instructions. The next day, the growth medium was replaced with fresh medium containing compounds of interest. Dimethyl sulfoxide (DMSO) was used as a vehicle. On the third day, the cells were harvested and processed for analyzing the luciferase activity using a GloMax Luminometer instrument (Promega) and the Dual-Luciferase Reporter Assay kit (cat. n. E1910 Promega, IT) [15].

### 4.4. MTT Assay

HEK293 cells were seeded at 2 × 10^3^ cells/cm^2^ density in 24-well plastic plates. One day after plating, CBM (up to 30 µM) was added to the culture medium for 24 h. Cell viability was evaluated with the 3-(4,5-dimethylthiazol-2-yl)-2,5-diphenyltetrazolium bromide (MTT; 0.5 mg/mL; Sigma-Aldrich) reduction assay, and formazan salt formation upon MTT reduction by the mitochondria of living cells was detected spectrophotometrically at 595 nm according to published procedures [41].

### 4.5. RNA Purification and Quantitative Real-Time PCR (qPCR)

Total RNA was isolated from cells using Pure Link^®^ RNA Mini Kit (Cat. N.: 12183018A; Thermo Fisher Scientific, Milan, Italy) following the manufacturer’s instruction, and then quantified by spectrophotometric analysis. The purified mRNA was reverse-transcribed using the iScript reverse transcriptase enzyme (Cat. N.: 1708840; Biorad, Milan, Italy). Quantitative real-time PCR was carried out in a CFX384 real-time PCR detection system (Bio-Rad, Milan, Italy) with specific primers (Table 1) using ssoAdvance Universal SYBR Green Supermix (Cat. N.: 1,725,270 Bio-Rad, Milan, Italy). Samples were amplified simultaneously in quadruplicate in a one-assay run with a non-template control blank for each primer pair to control for contamination or primer dimer formation, and the ct (cycle threshold) value for each experimental group was determined. Housekeeping genes (the ribosomal protein S16) were used as an internal control to normalize the ct values using the 2^−Δct^ formula [41].

### 4.6. Western Blotting Analysis

The 3T3-L1 cells were subjected to western blot analysis following the procedure previously described [41]. Briefly, following the treatment with palmitate, rosiglitazone, or CBM, differentiated 3T3-L1 cells were homogenized in lysis buffer composed of 1× Tris–HCl/NaCl/EDTA (TNE) buffer, 1% (*v*/*v*) Triton X-100, protease (cat. n. P8340, Sigma-Aldrich, MI Italy), and phosphatase (cat. n. P5726, Sigma-Aldrich, MI Italy) inhibitor cocktails at pH 7.4. Lysates were kept in orbital shaker incubator at 220 rpm at 4 °C for 30 min and then centrifuged for 15 min at 13,000× *g* at 4 °C. The supernatants were transferred to tubes and quantified by DC Protein Assay (Bio-Rad, Milan, Italy). Subsequently, the samples (60 μg of total protein) were heated at 70 °C for 10 min in NuPAGE LDS Sample Buffer (cat. n. NP0007, Life Technology, Milan, Italy) plus Sample Reducing Agent (cat. n. NP0004, Life Technology MI Italy) and loaded onto 4%–12% Bis-Tris Protein Gels (cat. n. NP0336PK2, Life Technology, Milan, Italy) and then transferred to a polyvinylidene fluoride (PVDF) membrane. The primary antibodies used were (a) Akt (9272, Cell Signaling, USA; diluted 1:1000); (b) phospho-Akt (Ser473) XP (4060, Cell Signaling, USA; diluted 1:2000). Reactive bands were detected by enhanced chemiluminescence (ECL-plus; Bio-Rad, Segrate, Italy). The intensity of bands was analyzed on a ChemiDoc station with Quantity-one software (Biorad, Segrate, Italy).

### 4.7. Glucose Uptake Assay

The glucose uptake in 3T3-L1 was measured using the commercially available kit Glucose Uptake-Glo™ Assay (cat. J1341) purchased from Promega (Milan, Italy) following the manufacturer’s indications. Briefly, 3T3-L1 were differentiated in 24-well culture plates following published procedures [23]. After 16 h of incubation with palmitate (plus Rosiglitazone or CBM), the cells were washed three times with PBS, and then stimulated with 100 nM insulin at 37 °C for 20 min [30]. The bioluminescent signal was acquired on a Glomax luminometer (Promega, Italy).

### 4.8. Chemical Reagents

Dimethyl sulfoxide (cat. n. W387520), sodium palmitate (cat. n. P9767), fenofibrate (cat. n. F6020), and insulin (cat. n. I6634) were from Sigma Aldrich (Milan, Italy); rosiglitazone (cat. n. 5325) was from Tocris Bioscience (Bristol, UK).

### 4.9. Statistical Analysis

Data are expressed as means ± S.E.M. of the given number of experiments (*n*). Datasets were compared by use of matched Student’s *t*-tests or, if necessary, with one-way analysis of variance, followed by Tukey’s test. Statistically significant differences were accepted at *p* ≤ 0.05.

## Figures and Tables

**Table 1:** Primers used in this study.

Gene	Forward Sequence (5′–3′)	Reverse Sequence (5′–3′)
***PPARγ***	GTCGGTTTCAGAAGTGCCTTG	GCTTTGGTCAGCGGGAAG
***FABP4***	TGTGATGCCTTTGTGGCAACCTG	TATGATGCTCTTCACCTTCCTGTCG
***C/EBP*** *α*	CAAGAACAGCAACGAGTACCG	GTCACTGGTCAACTCCAGCAC
***S16***	CTGGAGCCTGTTTTGCTTCTG	TGAGATGGACTGTCGGATGG
***GLUT4***	GCTCTGACGATGGGGAAC	CCAACACGGCCAAGACATTG
***FAS***	GGAGGTGGTGATAGCCGGTAT	TGGGTAATCCATAGAGCCCAG
***Adiponectin***	TGACGACACCAAAAGGGCTC	GAGTGCCATCTCTGCCATCA
